# Dr. William F. Elmendorf

**Published:** 1918-12

**Authors:** 


**Dr. William F. Elmendorf,** Buffalo 1890, died at his home in Buffalo, Sept. 28, of spinal meningitis, aged 64.

